# High attack rate for malaria through irregular migration routes to a country on verge of elimination

**DOI:** 10.1186/1475-2875-12-276

**Published:** 2013-08-06

**Authors:** Kolitha Wickramage, Risintha G Premaratne, Sharika L Peiris, Davide Mosca

**Affiliations:** 1 Health Unit, International Organization for Migration (IOM), No. 62, Green Path, Ananda Coomaraswamy Road, Colombo 3, Sri Lanka; 2 Anti-Malaria Campaign, Ministry of Health, No. 555/5, Public Health Building, Narahenpita, 5 Colombo, Sri Lanka; 3 Migration Health Department, International Organization for Migration (IOM), 17 Route des Morillons, 1211 Geneva 19, Switzerland

**Keywords:** Irregular migrants, Human smuggling, Malaria elimination

## Abstract

Irregular migration in the form of human smuggling and human trafficking is recognized as a global public health issue. Thirty-two cases of *Plasmodium falciparum* were detected in 534 irregular migrants returning to Sri Lanka via failed human smuggling routes from West Africa in 2012, contributing to the largest burden of imported cases in Sri Lanka as it entered elimination phase. Beyond the criminality and human rights abuse, irregular migration plays an important, but often forgotten, pathway for malaria re-introduction. Active surveillance of the growing numbers of irregular migrant flows becomes an important strategy as Sri Lanka advances towards goals of malaria elimination.

## Background

Sri Lanka is heralded as a ‘success story’ for malaria control in Asia having succeeded in reducing malaria cases by 99.9% since 1999 and is aiming to eliminate the disease entirely by 2014 [1]. This report focuses on a migrant flow of major importance for malaria importation that, until recently, has received little attention from public health authorities.

Since the end of the protracted civil conflict in 2009, there have been an unprecedented number of migrants leaving Sri Lanka to countries such as Australia, Canada and the UK via ‘irregular migration’ routes [2]. An irregular migrant is defined as someone who, owing to illegal entry or the expiry of his or her visa, lacks legal status in a transit or host country [3]. Irregular migration takes many forms, ranging from human smuggling to trafficking of persons for purpose of exploitation. Globally, numbers of undocumented cases have increased despite spending on enforcement measures at major destination countries [4]. A significant number of such migrants may remain stranded in transit or destination countries, often in clandestine situations or in detention facilities. In such situations, many have poor access to health services, become exposed to endemic diseases, and/or are vulnerable to violence, exploitation and other health risks [5]. The International Organization for Migration (IOM) estimates that 10-15% of the world’s total international migrant population of 214 million persons are irregular migrants [6]. IOM working with member states assists such stranded migrants to voluntarily return to their countries of origin through Assisted Voluntary Return and Reintegration (AVRR) programmes.

## Methods

### Screening strategy

From the end of 2011, local and international law enforcement authorities intercepted people-smuggling operations from Sri Lanka to Canada across nine West African nations: Togo, Benin, Guinea, Sierra Leone, Mali, Ghana, Senegal, and Mauritania. In close coordination and partnership with the Governments of Sri Lanka, Canada and West African nations, IOM assisted these irregular migrants who are intercepted or detained, to return to their place of origin.

From January to December 2012, all irregular migrants returning from West African countries were subjected to malaria screening upon arrival at the Bandaranayke International Airport (BIA) in Sri Lanka. Screening was conducted on site using the rapid diagnostic test kit CareStart™ Malaria HRP2/PLDH, with 98% sensitivity and 97.5% specificity for *Plasmodium falciparum*[7], and microscopic examination of blood smears, collected at the airport and performed at the national reference laboratory. Health personnel from the airport medical unit, Anti-Malaria Campaign (AMC) and IOM officials were involved in facilitating the on-arrival screening process. Under a directive of the Anti-Malaria Campaign, repeat RDTs were carried out for all returnees at district level within one week of their arrival at home destination. This intensive follow-up was carried out with the collaborative efforts of both the AMC and IOM field staff.

### Ethical consideration

Mandatory testing of all returnees were performed according to standard Ministry of Health Anti-Malaria Campaign Guidelines and routine protocols. All returnees were provided clear explanation on the testing at pre-departure phase and upon arrival before test was conducted.

## Results

Of the total number of returnees screened (n=534), 32 were positive for *P. falciparum*. Nearly two thirds (n=19) were identified at the point of entry at the BIA and 13 during district level follow-up. The total number of malaria cases from irregular migration routes in accounted for 76% (32/42) of the total number of *P. falciparum* cases detected in Sri Lanka in 2012. This route contributed to 46% (32/70) of the total number of imported malaria cases in the same year. Imported cases overtook indigenously acquired cases of malaria for the first time in Sri Lankan in 2012, contributing to three-quarters of the total malaria burden (70/93).

**Figure 1:**
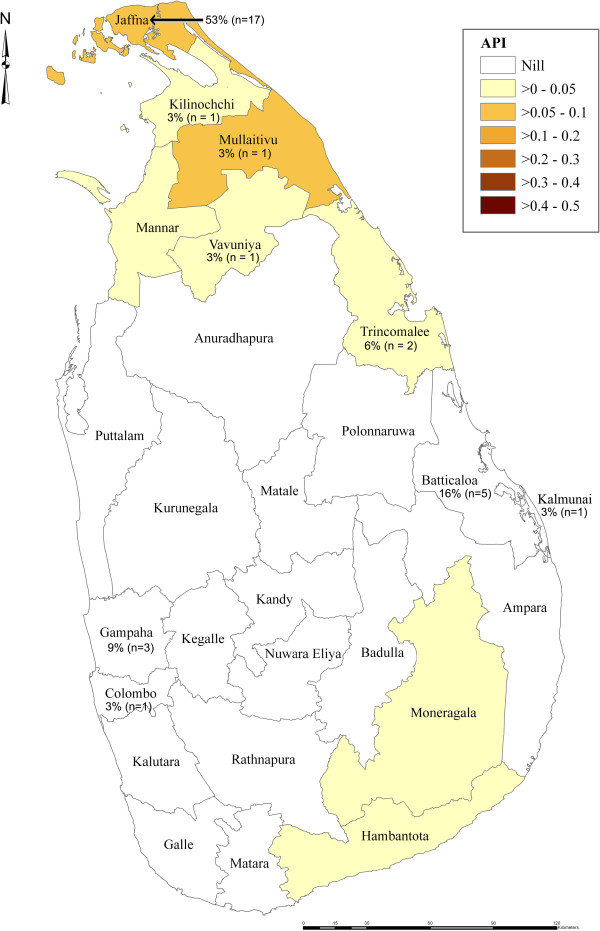
Map showing annual parasite incidence (API) (total number of positives cases per 1000 risk population) for the year 2002 superimposed with place of destination of returnee cases.

Figure 1 superimposes the districts of return of the irregular migrants with the geographical map of the Annual Parasite Index (total number of positives cases per 1,000 risk population) for Sri Lanka for the Year 2012. It shows that the largest number of irregular migrants (n=17) had returned to Jaffna district which has the highest API of >0.2 to 0.3 in comparison to other districts in Sri Lanka.

**Figure 2:**
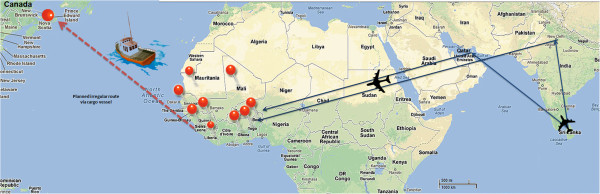
**Irregular migration routes from Sri Lanka to Canada via West Africa.** Blue indicates air routes and red markers represent the nine countries to which migrants entered before travelling via land routes to converge on a single port (Sierra Leone) to board a cargo vessel. Red dotted line represents the planned sea route. (Image developed by corresponding author. Map derived by Google maps).

Economic hardship, disenfranchisement and other social determinants form powerful push-factors for those marginalized to seek opportunities through irregular migration. The rational for such increased people movements from Sri Lanka are interlinked to complex social and political determinants, which warrants a detailed description beyond the scope of this research article. The largest group of returnees was from Benin (n=20, 77%), followed by Nigeria (9%), Guinea (13%), Liberia (6%), Togo (6%) and Sierra Leone (3%). Socio-demographic data revealed that the majority were males (91%), young (mean age 30 years), of Tamil ethnicity (94%), and originated from North and Eastern Provinces of Sri Lanka (88%). The average duration of stay in Africa was 20.5 weeks. Their prolonged stay in endemic settings increased the risk of transmission. Qualitative assessments (through return interviews) revealed a number of persons had suffered febrile illnesses during their stay in West Africa. However, details on total number, time and place could not be characterized through such narrative construction. It was also revealed that smugglers used force and intimidation to prevent the migrants from escaping. The smugglers intended to channel all cohorts of migrants to a single port (Sierra Leone), and then charter a large fishing vessel to enter Canada illegally (Figure 2).

## Discussion

Malaria incidence in returnees from source countries have proven to be a sensitive predictor of malaria risk, particularly where there is sub-national transmission [8]. The fact that the largest number of migrants returned to districts with the highest API indexes reported nationally is also significant. Re-introduction and risk of spreading the parasites occurs when there is a long-term return into areas of endemicity with presence and prevalence of the mosquito vector. For this reasons the close follow up and monitoring performed by the AMC and IOM field based teams is an important strategy. Unlike other categories of inbound migrants, such as tourists, who may also import malaria to the country, returning Sri Lankan citizens from endemic areas are more likely to be exposed to mosquito bites and hence are more likely to contribute to the spread of malaria upon return to their homes within locally endemic regions. Other inbound migration categories include: returning Sri Lankan labour migrant workers, Sri Lankan armed forces personnel from UN peace keeping missions, and returning students.

The attack rate for malaria in this migrant group using irregular modes of travel is considerably high (sixty cases per 1,000), when compared to the risk of contracting malaria for regular travellers returning from West Africa at three per 1,000 [9]. For the migrants themselves, their ‘illegal’ status and clandestine nature of movements enhanced health vulnerability, including having little or no access to health care in transit countries. Remarkably, 98% of irregular migrants from West Africa had undertaken yellow fever vaccinations at the Ministry of Health vaccination centre in Colombo prior to their departure (proven through receipt of vaccination card). The people-smugglers were aware of International Health Regulation (IHR) checks at ports of entry, and insisted these be obtained by the migrants during pre-departure phase.

An analysis of registry data on yellow fever vaccinations of Sri Lankan travellers from 1998 to 2011. Since the end of conflict in 2009, there has been a rapid increase in the volume of travellers to malaria-endemic countries, with the majority (97% of the 4,500) departing to West Africa.

## Conclusions

Irregular migration will always exist in a globalized world of increasing disparity and criminal opportunism. The post-conflict period has seen a dramatic increase in the number of irregular migrant flows from Sri Lanka [10]. Surveillance of inbound migrant flows from endemic areas is vital to prevent the re-emergence of disease, especially as Sri Lanka has entered the malaria elimination phase.

Beyond the challenge of combating the criminal networks, abuse and the exploitative practices of people smugglers, irregular migration plays an important but often forgotten pathway for malaria re-introduction. More attention is needed by global public health communities to the contribution and dynamics of malaria importation and introduction via irregular migrant routes.

## Competing interests

The authors declare that they have no competing interests. No financial assistance has been provided in undertaking this research.

## Authors’ contributions

KW: the conception and design of the paper; KW and SP: involved in acquisition of data, analysis and interpretation of data; KW, RP and SP: drafting the article or revising it critically for important intellectual content; KW, SP and DM made final approval of the version to be submitted. The final manuscript has been approved by all authors.
